# SATB1 Makes a Splash in T Cell Wnt Signaling

**DOI:** 10.1371/journal.pbio.1000295

**Published:** 2010-01-26

**Authors:** Caitlin Sedwick

**Affiliations:** Freelance Science Writer, San Diego, California, United States of America

For microbial invaders to establish an infection, they must overcome the defenses mounted by one of the body's staunchest defenders, the T cell. T cells come in several different flavors, each with a specific role to play. Cytotoxic T cells battle intracellular pathogens, while helper T cells (Th cells) direct antibody production and orchestrate the overall immune response by secreting intercellular messaging proteins known as cytokines. Th cells can differentiate further into two major types (known as Th1 or Th2 cells), which can be distinguished from one another based on which cytokines they secrete.

In order to mount an effective immune response, T cells of all kinds must be able to recognize foreign invaders while also ignoring body-derived materials. Therefore, before they differentiate into cytotoxic T cells or Th cells, immature T cells (known as thymocytes because they are found in the thymus) go through a series of checkpoints to ensure they fulfill these requirements. During this lengthy checking process, thymocytes require external signals to help support their growth. One of these support signals is provided by Wingless (Wnt) proteins. First characterized in flies as a mutation that impairs thoracic segment development, Wnt signaling works similarly in all organisms; when soluble Wnt proteins bind to their receptors on cells, they initiate a signaling cascade that ultimately results in the accumulation of the protein β-catenin in the cells' nucleus. There, β-catenin binds to and activates other proteins (known as transcription factors) that control the expression of genes needed for cell growth. In this issue of *PLoS Biology*, Dimple Notani, Sanjeev Galande, and colleagues provide novel insights into how the transcription factor Special AT-rich Binding Protein 1 (SATB1), known to be required for T cell development, affects the outcome of β-catenin signaling at the transcription level. They show how β-catenin and SATB1 interact in T cells to affect Th cell growth and differentiation.

SATB1 is expressed primarily in thymocytes and in Th2 cells. It is known to bind to DNA, tethering DNA to the protein scaffolding that lines the nucleus. This helps organize DNA into tidy loops for better control of gene expression. SATB1 also serves as a molecular adaptor for several other proteins that work to pack DNA into an inactive state. In this way, SATB1 is thought to repress the activity of genes found nearby its DNA binding sites. However, some of the genes SATB1 represses are known to be upregulated by Wnt signals – a fact that led Notani and colleagues to explore whether (and how) SATB1 and Wnt signaling might interact in T cells.

When the authors examined the nuclei of Wnt-stimulated thymocytes under a microscope, they found that SATB1 and β-catenin (the major nuclear component of Wnt signaling) are present in the same parts of the nucleus, suggesting these two proteins might directly interact. Subsequent experiments showed that SATB1 and β-catenin are not just in proximity to each other, but actually bind directly to one another: the first half of SATB1 protein binds to β-catenin, while the last part of β-catenin binds to SATB1.

The finding that SATB1 and β-catenin bind to one another led the researchers to examine whether Wnt signaling might cause SATB1 to recruit β-catenin to genes. They found that Wnt signaling causes an increase in SATB1 DNA binding by promoting the deacetylation of SATB1—a modification that is known to increase SATB1's affinity for DNA. What's more, this increased SATB1 binding to genes is mirrored by increased levels of β-catenin on those same genes. Collectively, these data suggest that Wnt signaling increases SATB1 binding to DNA, and that SATB1 then recruits β-catenin to DNA. Once β-catenin is bound to SATB1, β-catenin can recruit additional partners to help stimulate gene expression, thereby indirectly converting SATB1 from a repressor to an activator of gene expression.

The authors' findings add an important new detail to our understanding of the role played by both SATB1 and Wnt in thymocytes. But, because SATB1 is specifically expressed in Th2 cells, Notani and colleagues also wanted to explore what contributions SATB1 (and Wnt signaling) might make to Th2 cell differentiation. They found that Th cells of all kinds make Wnt proteins and so can stimulate themselves with Wnt signals. However, Th2 cells experience higher levels of Wnt signaling than do Th1 cells. Therefore, in Th2 cells, SATB1 and β-catenin can cooperate to promote the expression of GATA3, another transcription factor that is known to control the differentiation and development of Th2 cells, thereby promoting the production of Th2-characteristic cytokines. These new insights will allow researchers to better understand how T cell development is controlled by chromatin organizing factors in collaboration with various signaling processes, how this process can go awry, and the diseases that can result from T cell–related immune disorders (including autoimmunity or immune deficiencies).

**Notani D, Gottimukkala KP, Jayani RS, Limaye AS, Damle MV, et al. (2010) Global Regulator SATB1 Recruits β-Catenin and Regulates T_H_2 Differentiation in Wnt-Dependent Manner. doi:10.1371/journal.pbio.1000296**

